# Crystal structure of a thermophilic fungal cyanase and its implications on the catalytic mechanism for bioremediation

**DOI:** 10.1038/s41598-020-79489-3

**Published:** 2021-01-11

**Authors:** Bibhuti Ranjan, Philip H. Choi, Santhosh Pillai, Kugenthiren Permaul, Liang Tong, Suren Singh

**Affiliations:** 1 Department of Biotechnology and Food Science, Faculty of Applied Sciences, Durban University of Technology, Durban, 4000 South Africa; 2 Department of Biological Sciences, Columbia University, New York, NY 10027 USA

**Keywords:** Biocatalysis, Structural biology, Environmental sciences

## Abstract

Cyanase catalyzes the bicarbonate-dependent degradation of cyanate to produce ammonia and carbon dioxide, and ammonia is a considerable alternative nitrogen source. Strikingly, the cyanase from the thermophilic fungus *Thermomyces lanuginosus* (Tl-Cyn) has the highest catalytic efficiency reported among these enzymes. However, its molecular mechanism of action is not clearly understood, because currently there is no structural information available on fungal cyanases. Here we report the crystal structure of Tl-Cyn in complex with inhibitors malonate and formate at 2.2 Å resolution. The structure reveals extensive interactions at the subunit interfaces in a dimer, and a decamer is formed by a pentamer of these dimers. Our biochemical, kinetic and mutagenesis studies confirm the structural observations on the complex and provide further insights into its catalytic mechanism and inhibition. The structure has also aided the creation of a mutant enzyme with enhanced catalytic activity, and such enzymes may have the potential for biotechnological applications, including biotransformation and bioremediation. Moreover, other fungal cyanases with potentially high catalytic activity could also be predicted based on the Tl-Cyn structure, as the active site region among fungal cyanases are highly conserved.

## Introduction

Cyanide is one of the most toxic chemicals widely used in mining industries for the extraction of metals^1^. Cyanide is also applied as an anticaking agent in road salt and fire retardants, where several hundred tonnes of cyanide is released into the environment annually^2,3^. Cyanate is an important cyanide derivative formed by the oxidation of cyanide^1^. Moreover, it is also generated spontaneously from urea and carbamoyl phosphate^4,5^. The toxicity of cyanate possibly arises from its reactivity with amino, sulfhydryl, carboxyl, phenolic hydroxyl, imidazole, and phosphate groups in proteins^6^. Additionally, cyanate is widely used to inhibit physiological reactions in plants as a herbicide^7^ and in mammals as an uremic toxin^8^. In contrast, cyanate has been assumed to serve as a nitrogen source for the growth of certain microorganisms (having a functional cyanase) under nitrogen limitation^9,10^, while microbes lacking a cyanase gene are not able to grow in the presence of cyanate^11,12^.

Cyanase (EC 4.2.1.104; also known as cyanate hydratase and cyanate lyase) catalyzes the decomposition of cyanate into ammonium and CO_2_. Cyanases are found in bacteria^13–16^, fungi^17^ and plants^18^, where they have important roles for nitrogen assimilation or cyanate detoxification. Despite these important functions, the production of this enzyme is low in most known organisms^16–19^. Although the cyanase from bacteria is characterized in detail and its structure is also known^20,21^, no attempt has been made to enhance its production.

*Thermomyces lanuginosus*, a thermophilic fungus, has been known to produce the highest amount of xylanase and it also produces several other hydrolytic enzymes^22^. In addition, it has a ubiquitin degradation pathway which plays an essential role in responses to various stress, such as nutrient limitation, heat shock, and heavy metal exposure^23^. Owing to these requisite properties, this fungus has been identified as one of the organisms of choice for industrial applications. Furthermore, whole genome sequencing and secretome analysis of *T. lanuginosus*^23,24^ unexpectedly revealed the presence of a cyanase (Tl-Cyn). We have successfully over-expressed Tl-Cyn and evaluated its potential in cyanate detoxification^25–27^. Notably, this cyanase (Tl-Cyn) showed ~ 250-fold higher catalytic activity compared to other cyanases^25^, suggesting that it could be used for large-scale applications.

To get a better understanding of Tl-Cyn function and a deeper insight into its molecular mechanism requires structural information on this enzyme. To date, biochemical and structural studies have been limited mainly to bacterial cyanases and no structural information is available on fungal cyanase. We report here the crystal structure of the cyanase from *T. lanuginosus* (Tl-Cyn) at 2.2 Å resolution. The structure reveals extensive interactions at the subunit interfaces in a dimer, and a decamer is formed by a pentamer of these dimers. The structure of the monomer and the overall architecture of the decamer show substantial differences to those of a bacterial cyanase^20^. We have also determined the binding modes of substrate-analog inhibitors in the active site of Tl-Cyn and characterized their inhibition of the enzyme by kinetic studies, which provide molecular insights into Tl-Cyn catalysis. In addition, we found that mutations in the active site region of Tl-Cyn can reduce enzyme activity. Furthermore, we found that the Y14A mutant has higher catalytic activity compared to the wild-type, due primarily to an increase in *k*_cat_.

## Results

### Overall structure of Tl-Cyn

To gain insight into the Tl-Cyn structure, activity and enable its rational design, we have determined the crystal structure of Tl-Cyn at 2.2 Å resolution. The full-length *Tl-Cyn* gene was expressed in *E. coli* BL21 Star (DE3) cells and purified (Supplementary Fig. S1a). The stability of the purified Tl-Cyn was evaluated using the thermal shift assay, and the T_m_ of the protein was ~ 65 °C (Supplementary Fig. S1b). The refined structure has excellent agreement with the crystallographic data and the expected bond lengths, bond angles, and other geometric parameters (Table 1). A total of 99.4% of the residues are in the favored region of the Ramachandran plot, and none are in the disallowed region.

**Table 1:** Crystallographic data collection and refinement statistics.

Structure	Tl-Cyn
**Data collection**	
Space group	*P*2_1_2_1_2_1_
Cell dimensions	
a, b, c (Å)	75.37, 157.64, 163.87
α, β, γ (°)	90, 90, 90
Resolution (Å)	50.00–2.20 (2.28–2.20)*
*R*_merge_	9.6 (38.1)
Completeness (%)	99.90 (99.50)
Redundancy	4.4 (4.3)
**Refinement**	
Resolution (Å)	50.00—2.20 (2.28 – 2.20)
*R*_work_/*R*_free_	15.44 / 20.18
R.m.s deviations	
Bond lengths (Å)	0.018
Bond angles (°)	1.9
Ramachandran plot statistics	
Most favoured regions (%)	99
Additionally allowed regions (%)	1
Outliers (%)	0.0
PDB entry code	6XGT

*Highest resolution shell is shown in parenthesis.

**Figure 1:**
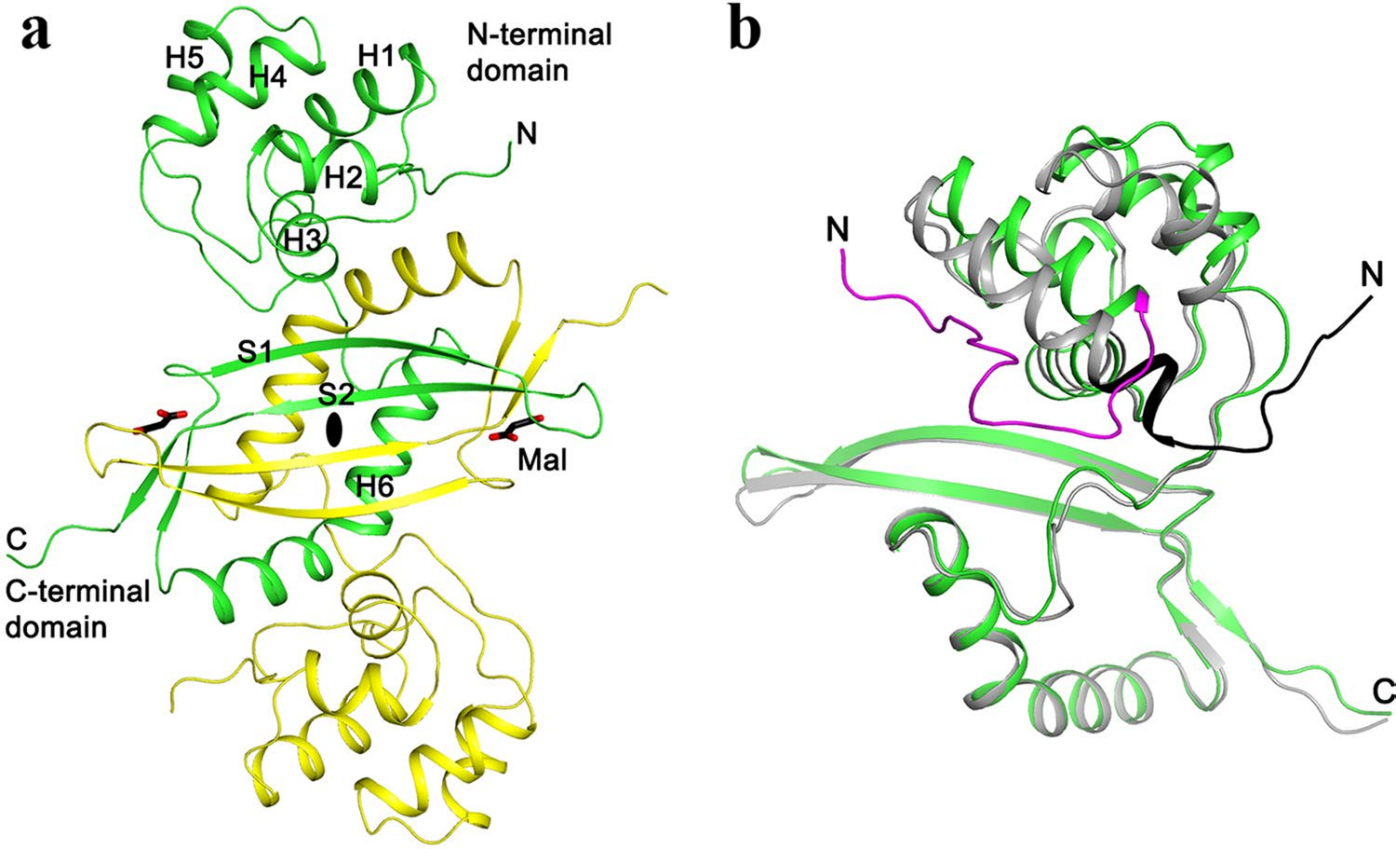
Structure of fungal cyanase dimer. (**a**) An intertwined C-terminal domain (residues 92–160) in the dimer structure of *T. lanuginosus* cyanase (Tl-Cyn). The two monomers are colored in yellow and green, and the two-fold axis of the dimer is indicated by the black oval. The two malonate molecules bound to the active sites of the dimer are shown as sticks, colored according to atom types (carbon black, and oxygen red) and labeled Mal. Each monomer contains six α-helices and two anti-parallel β-strands, labeled as H1–H6 and S1–S2, respectively. (**b**) Overlay of the structure of Tl-Cyn monomer (green) with that of *E. coli* cyanase (gray). Large structural differences are seen for the N-terminal domain. Especially, the N-terminal segments run in opposite directions in the two structures and are highlighted in magenta and black. The structure figures were produced using PyMOL (http://www.pymol.org).

The structure of cyanase contains an N-terminal α-helical domain (residues 1–91; helices H1–H5) and a C-terminal α + β domain (residues 92–161; two antiparallel β-strands S1–S2 and a helix H6) (Fig. 1a). There are extensive interactions between the C-terminal domains of two subunits of a dimer, which together form a four-stranded anti-parallel β-sheet that is flanked on one face with the two H6 helices (Fig. 1a).

**Figure 2:**
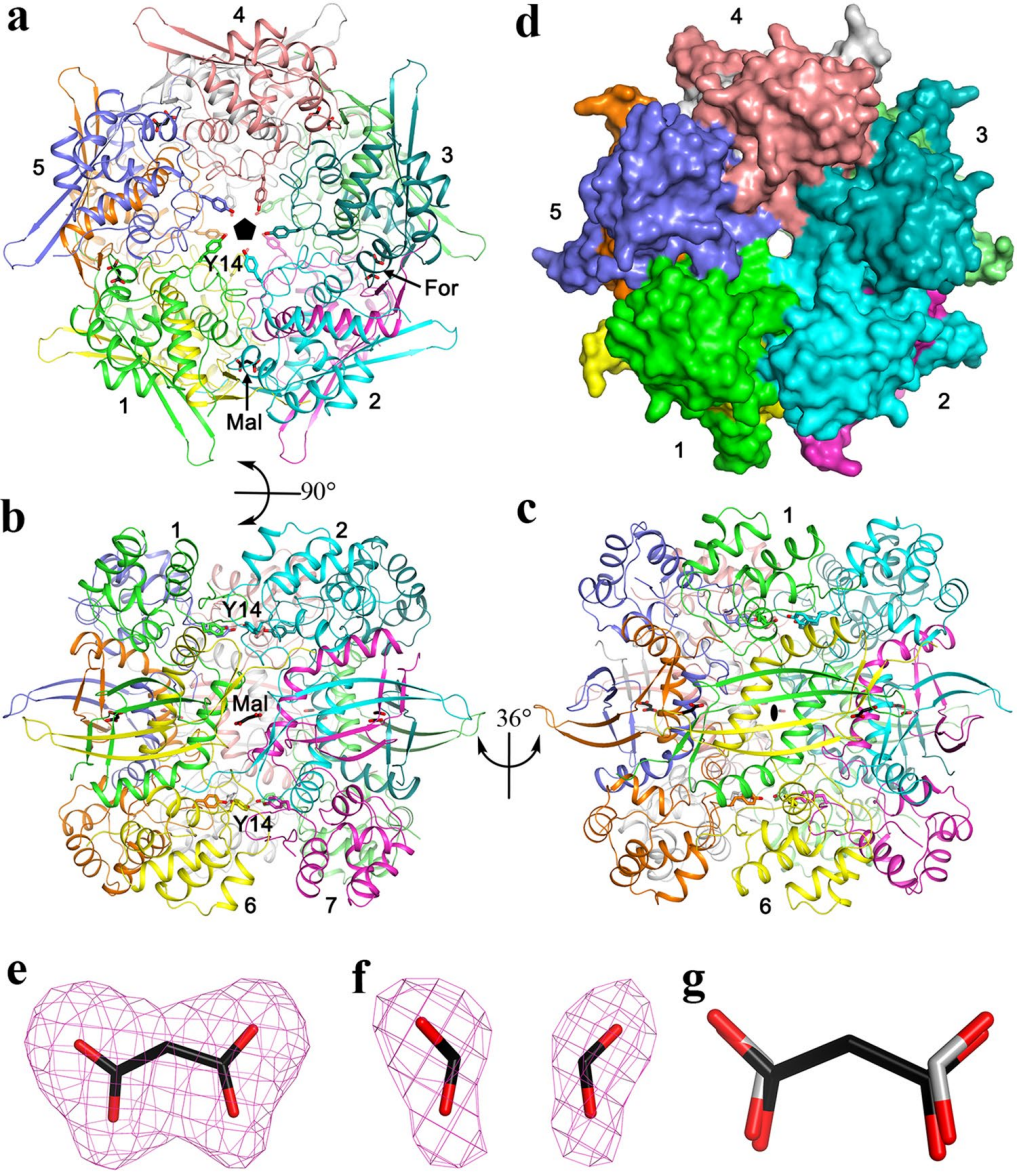
Crystal structure of the fungal cyanase decamer. (**a**) Overall structure of the Tl-Cyn decamer (top view). Each monomer is depicted in a different color. The side chains of Y14 are shown as sticks, pointing towards the center of the structure. The malonate (labeled Mal) and formate (labeled For) molecules bound to the active sites of the decamer are shown as sticks. The five-fold symmetry axis of the decamer is indicated with the black pentagon. (**b**) Overall structure of the Tl-Cyn decamer viewed after a 90° rotation around the horizontal axis. (**c**) Overall structure of the Tl-Cyn viewed after a 36° rotation around the vertical axis from panel (**b**). A two-fold axis of the decamer is indicated with the black oval. (**d**) Molecular surface of the Tl-Cyn decamer, viewed in the same orientation as in (**a**). (**e**) Omit F_o_ − F_c_ electron density at 2.2 Å resolution for malonate, contoured at 3 s. (**f**) Omit F_o_ − F_c_ electron density at 2.2 Å resolution for two formate molecules, contoured at 3σ. **g,** Overlay of the binding modes of malonate (black carbon atoms) and two formate molecules (gray carbon atoms).

A decamer of Tl-Cyn is formed by a pentamer of these dimers, with intimate contacts among them (Fig. 2a–d). The interfaces in this decamer primarily involve the N-terminal domain and the H6 helix, while the four-stranded β-sheet in the center of the dimer interface is located near the ‘equator’ of the decamer. There is a small channel through the entire decamer, along its five-fold symmetry axis (Fig. 2d).

The structure of Tl-Cyn shows substantial differences to that of *E. coli* cyanase^20^ (Fig. 1b), even though the two proteins share 40% sequence identity. With the C-terminal domains of the two structures in overlay, large differences in the positions of the helices in the N-terminal domain are observed. In particular, the N-terminal segment of the two enzymes run in nearly opposite directions. On the other hand, the overall appearance of the two decamers is similar (Supplementary Fig. S2a,b).

### Active site of Tl-Cyn

**Figure 3:**
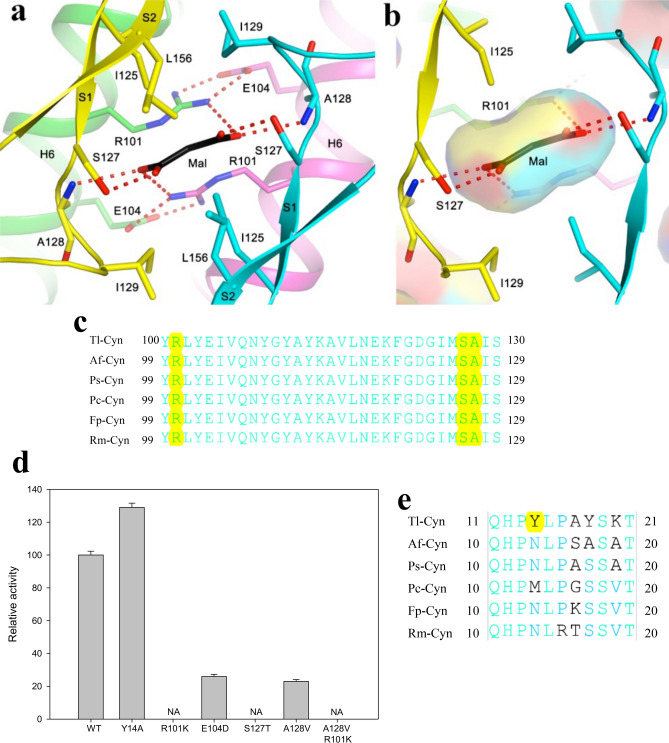
Structural insights into the catalytic mechanism of Tl-Cyn. (**a**) Schematic drawing of the detailed interactions between Tl-Cyn and the malonate molecule bound in the active site (sticks model and labeled Mal). Hydrogen-bonding interactions with the malonate are indicated with dashed lines (red). (**b**) Close-up view to that in (**a**), and the internal cavity for malonate is shown with semi-transparent surface. (**c**) Conserved sequence near the active site region of the C-terminal domain in fungal cyanases are shown in cyan. Residues that interact with Mal are shown with yellow background. Tl: *Thermomyces lanuginosus*, Af: *Aspergillus flavus*, Ps: *Penicillium subrubescens*, Pc: *Phaeomoniella chlamydospora*, Fp: *Fonsecaea pedrosoi*, Rm: *Rhinocladiella mackenziei*. (**d**) Catalytic activities of wild-type (WT) and mutant Tl-Cyn. The cyanate concentration is at 2 mM. The error bars represent the standard deviation from three independent measurements. NA, no activity observed under the condition tested. (**e**) Sequence alignment for the N-terminal domain of the fungal cyanases. Conserved residues are in cyan and a unique Y14 residue in Tl-Cyn is shown with yellow background.

The active site of Tl-Cyn is located in the C-terminal domain (Fig. 1a), at the interface between neighboring dimers of the decamer (Fig. 2a). This suggests that Tl-Cyn needs to be a decamer to be active. Malonate (Mal) is situated on a two-fold symmetry axis in some of the dimers of the decamer, while two formates (For) are observed in the other dimers (Fig. 2a). Both Mal and For are present in the Tacsimate solution used to crystallize Tl-Cyn and have good electron density (Fig. 2e,f). The carboxylate groups of Mal are recognized by six hydrogen bonds from residues of four monomers (Fig. 3a,b), and formate has the same interactions as it has essentially the same binding mode as the carboxylates of malate (Fig. 2g). Specifically, the carboxylate is hydrogen-bonded to the side chain of Ser127 and the main-chain amide of Ala128 from one monomer (Fig. 3a). One of the carboxylate oxygen atoms is also hydrogen-bonded to the side chain of Arg101 from another monomer, which is also involved in a bidentate ion-pair with Glu104 from a third monomer. The carboxylate group of For is recognized in a similar fashion.

Residues in the active site region of Tl-Cyn are highly conserved among fungal cyanases, sharing 100% amino-acid sequence identity (Fig. 3c). Thus, elucidation of the Tl-Cyn structure could be beneficial to improve the catalytic properties of fungal cyanases and also allow for the creation of novel enzymes for biotechnological applications.

### Mutagenesis studies to provide insights into the catalytic mechanism

We designed a series of point mutations that are expected to perturb the hydrogen-bonding interactions with Mal based on the structural observations and evaluated their effects on catalysis (Fig. 3d). When the arginine (R101) and serine (S127) residues (in Tl-Cyn) were mutated to lysine and threonine, respectively, the catalytic activity was completely inhibited (Fig. 3d). We also found that the A128V and E104D mutations greatly reduced the catalytic activity by 77 and 74%, respectively, compared to the wild-type (Fig. 3d). The Glu104 residue is not directly involved in the hydrogen-bonding interactions with Mal; however, it stabilizes the catalytic residue Arg101, which resulted in the loss of catalytic activity. We also observed that, the A128V/R101K double mutation completely eliminated the catalytic activity of the enzyme. These data strongly support the structural observation on the active site of Tl-Cyn in which Arg101, Ser127, and Ala128 residues are involved in the interaction with inhibitor (Mal) residue by hydrogen bonds (Fig. 3a).

In addition, a unique feature of Tl-Cyn is the Tyr14 residue (Fig. 3e), which is located in the center of the structure and helps to reduce the size of the central channel (Fig. 2a,c). Surprisingly, the Y14A mutant displayed ~ 1.3-fold higher catalytic activity as compared to the wild-type Tl-Cyn (Fig. 3d). This could be due to the bulky side chain of Tyr14 residue sterically hindering substrate access to and/or product release from the active site of the Tl-Cyn, while mutating Tyr14 to a smaller amino acid (Ala) might have facilitated more substrate binding to the active site of the mutant. Because of the structural differences in the N-terminal segment (Fig. 1b), the bacterial enzyme does not have an equivalent to Tyr14, and the residue closest to Tyr14 in the structure is Asn9.

We next used kinetic experiments to further characterize the effects of mutations on the catalytic efficiency (*k*_cat_/*K*_m_) of Tl-Cyn. We observed that the catalytic efficiency of the E104D and A128V mutants was 2.79 ± 0.025 × 10^7^ s^−1^ M^−1^ and 2.93 ± 0.12 × 10^7^ s^−1^ M^−1^, which is ~ 3.5 and ~ 3.3-fold lower than that for the wild-type enzyme, respectively (Table 2). In contrast, the catalytic efficiency of the Y14A mutant was 12.04 ± 0.69 × 10^7^ s^−1^ M^−1^, which is ~ 1.2-fold higher compared to the wild-type enzyme (9.8 ± 0.4 × 10^7^ s^−1^ M^−1^), due mostly to a higher *k*_cat_.

**Table 2:** Summary of kinetic data for wild-type and mutant cyanases.

Protein	*k*_cat_ (s^−1^)	*K*_m_ (mM) (for cyanate)	*k*_cat_/*K*_m_ (s^−1^ M^−1^)
Wild-type	3.56 ± 0.19 × 10^4^	0.36 ± 0.04	9.8 ± 0.4 × 10^7^
Y14A	4.81 ± 0.11 × 10^4^	0.40 ± 0.01	12.04 ± 0.69 × 10^7^
R101K	NA^α^	NA^α^	NA^α^
E104D	5.84 ± 0.026 × 10^3^	0.21 ± 0.003	2.79 ± 0.025 × 10^7^
S127T	NA^α^	NA^α^	NA^α^
A128V	5.72 ± 0.029 × 10^3^	0.20 ± 0.007	2.93 ± 0.12 × 10^7^
A128V/R101K	NA^α^	NA^α^	NA^α^

^α^No activity was observed under the same conditions.

### Malonate and formate are cyanase inhibitors

**Figure 4:**
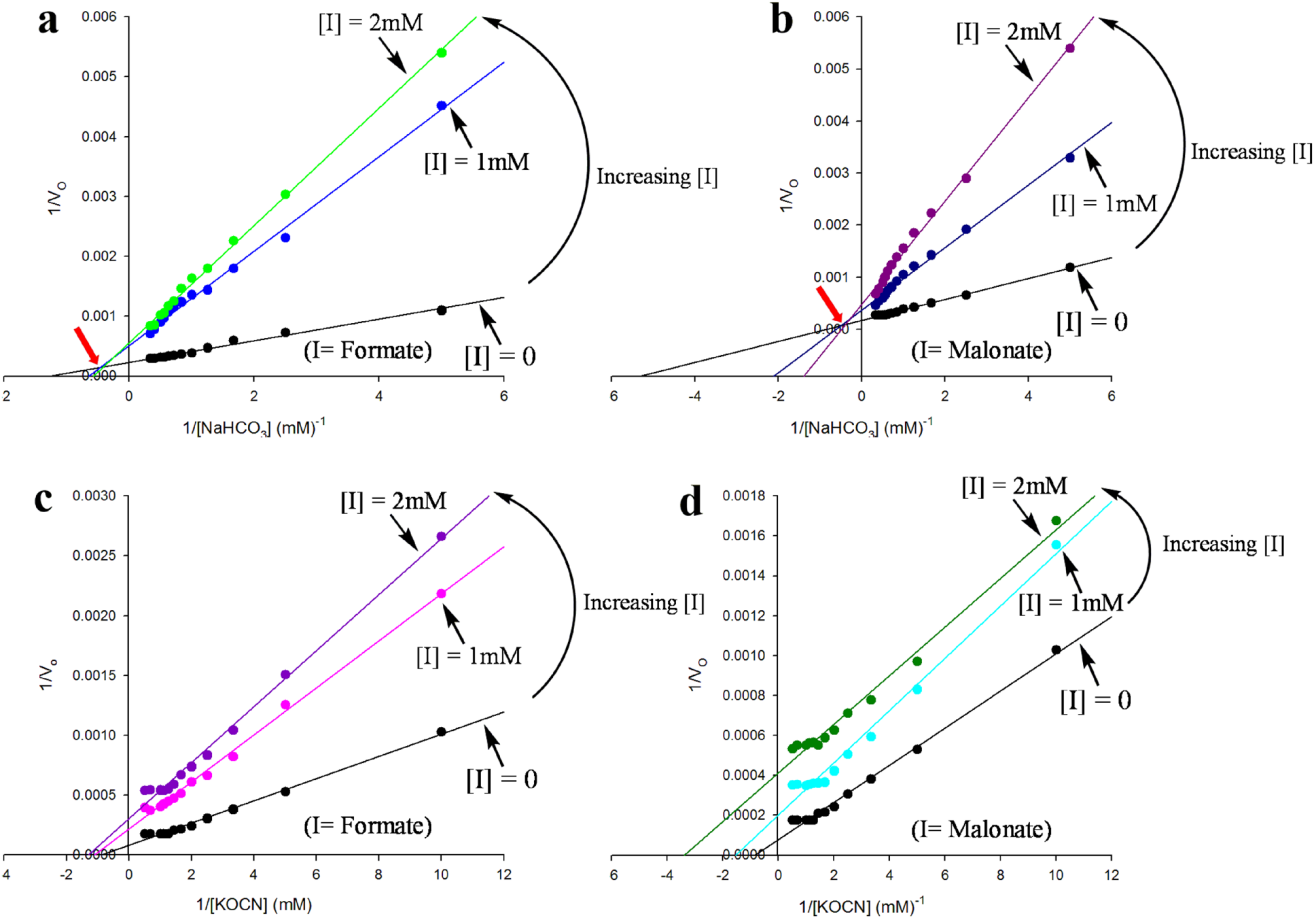
Kinetic studies on the inhibition of Tl-Cyn by malonate and formate. (**a**,**b)** A set of double-reciprocal plots, one obtained in the absence of inhibitor and two at different concentrations of inhibitor, formate and malonate, respectively in the presence of varying concentrations of NaHCO_3_. The location of the intersection point of the lines is indicated with the red arrow. (**c**,**d)** A set of double-reciprocal plots, one obtained in the absence of inhibitor and two at different concentrations of inhibitor, formate and malonate, respectively in the presence of varying concentrations of KOCN. Data points are means taken from three representative set of experiments.

In order to confirm the structural observations that malonate and formate are present in the active site of Tl-Cyn (Fig. 2a), kinetic experiments were performed to determine the inhibition mechanisms. The data are plotted in the form of 1/v_0_ against 1/[S], where v_0_ is the initial velocity of the enzyme-catalyzed reaction (µmoles mg^−1^ min^−1^) and [S] is the substrate concentration (mM) (Fig. 4). Based on the double-reciprocal plots, formate and malonate have direct inhibitory effect on the catalysis of Tl-Cyn^28^. Furthermore, the point of intersections observed in the double-reciprocal plots showed that it is a mixed-type of inhibition with respect to bicarbonate HCO_3_^−^ (Fig. 4a,b). In contrast, no point of intersections were observed with respect to cyanate OCN^−^, and the presence of parallel lines in the double-reciprocal plots are indicative of the uncompetitive-type of inhibitions (Fig. 4c,d). The inhibitory constants (*K*_i_) for malonate and formate are in the low mM range (Table 3). Our kinetic results confirmed that formate and malonate could inhibit the catalytic activity of Tl-Cyn, which further support the structural observations. Overall, mutagenesis and kinetic studies confirmed the presence of malonate and formate inhibitors at the active site of the Tl-Cyn. We propose a possible mechanism for the decomposition of cyanate and kinetic inhibition of the Tl-Cyn (Supplementary Fig. S3a,b). A similar mechanism for the decomposition of cyanate has been proposed the kinetic studies of another cyanase^14^.

**Table 3:** Summary of inhibition kinetics of Tl-Cyn.

Substrate	Inhibitor	Type of inhibition	Inhibitory constant (K_i_) (mM)	
			K_ia_^β^	K_ib_^γ^
KOCN	Formate	Uncompetitive	–	0.70 ± 0.06
KOCN	Malonate	Uncompetitive	–	0.54 ± 0.12
NaHCO_3_	Formate	Mixed	0.41 ± 0.11	1.19 ± 0.39
NaHCO_3_	Malonate	Mixed	0.23 ± 0.01	1.08 ± 0.20

^β^The K_i_ for binding to the free enzyme.

^γ^The K_i_ for binding to the enzyme–substrate complex.

## Discussion

Rapid industrialization and proliferative development of mining industries have resulted in increased global pollution and environmental deterioration, due to the release of toxic chemicals such as cyanide or cyanate^29,30^. Although the role of Tl-Cyn in the bioremediation of cyanurated-waste is known^25–27^, the molecular mechanism by which Tl-Cyn carries out decomposition of cyanate is unclear. The structure of the inhibitor-bound Tl-Cyn has elucidated the molecular mechanism of enzyme catalysis, in which the inhibitor interacts with the catalytic residues of the Tl-Cyn and explains the binding affinity between them (Fig. 3 a,b). In particular, it also provides a better understanding for the interpretation of the kinetic data.

Based on results from our mutagenesis studies, it is clear that Arg101 and Ser127 are crucial for the catalytic activity of Tl-Cyn, as highlighted by our structural observations (Fig. 3a,b). This is supported by a previous report wherein it has been shown that Ser122 (equivalent to Ser127) is responsible for the binding of substrates (OCN^-^ and HCO_3_^−^) and guanidinium group of the Arg96 (equivalent to Arg101) stabilizes the negative charge of the substrates^20^. In addition, we performed structure-based protein engineering by replacing Tyr14 (a residue located in the center of structure) to Ala. The Y14A mutant showed higher catalytic activity and the structure also indicates a possible mechanism for the enhanced catalytic activity. This is a key finding as the structure-based engineering of a Tyr14 residue (N-terminal), which is located away from the catalytic site (C-terminal), gives rise to increased cyanase activity. Further mutagenesis studies are needed to enhance the substrate specificity of fungal cyanases, and our structure would provide insights on the effect of mutations with respect to substrate binding.

Our kinetic studies also corroborate the presence of inhibitor molecules viz*.*, malonate and formate at the active site of Tl-Cyn, which is in agreement with the structural observations. Although there is no direct inhibition (uncompetitive-type) observed with respect to cyanate by these inhibitors, it however showed competitive- as well as uncompetitive-type inhibition with respect to bicarbonate. Further, it has been shown that bicarbonate is necessary for the binding of cyanate to the active site of cyanase for its degradation^12^, hence, inhibition of bicarbonate leads to the inhibition of cyanate binding at the active site of Tl-Cyn. It has also been observed that bicarbonate and cyanate have a competitive relationship for a catalytic binding site^12^. In addition, inhibition of cyanase activity by malonate in *E. coli* was also previously reported^31^, which supports our findings. Our structural analysis would therefore be useful for protein engineering to design biocatalysts with enhanced catalytic properties, which would be valuable for industries focused on sustainable bio-remediation.

## Methods

### Protein expression and purification

The *Tl-Cyn* gene was sub-cloned from the previous clone^25^, into the pET28a vector (Novagen), using *Nde*I and *Xho*I restriction sites, which introduced an N-terminal hexa-histidine tag. The recombinant protein was over-expressed in *E. coli* BL21 Star (DE3) cells (Novagen). The cell culture was grown in LB medium at 37 °C with shaking until the OD_600_ reached ~ 0.6, which was induced with 0.8 mM IPTG and allowed to grow at 20 °C for 16 h. The culture was then harvested by centrifugation and cells were resuspended in lysis buffer, consisting of 20 mM Tris–HCl (pH 8.0), 250 mM NaCl and lysed by sonication. The soluble protein was bound to nickel-charged immobilized-metal affinity resin (Qiagen) and eluted using lysis buffer supplemented with 250 mM imidazole. The protein was further purified by gel filtration chromatography (Sephacryl S-300, GE Healthcare)^32^ using buffer consisting of 20 mM Tris–HCl (pH 8.0) and 150 mM NaCl. The purified protein was concentrated using10 kDa ultrafiltration membrane cartridge (Millipore) to 13 mg/mL in a buffer containing 20 mM Tris–HCl (pH 8.0) and 150 mM NaCl, flash-frozen in liquid nitrogen and stored at − 80 °C. The N-terminal hexa-histidine tag was not removed for crystallization.

### Thermal shift assay

Thermal stability of the Tl-Cyn at 10 µM concentration was analyzed at various temperatures using the Mx3005P Real-Time PCR system (Stratagene). Tl-Cyn was mixed with the fluorescence dye (SYPRO orange; Invitrogen) for monitoring protein unfolding. All assays were performed in duplicate and contained final concentrations of 20 mM Tris–HCl (pH 8.0) and 150 mM NaCl. The temperature was increased from 25 to 99 °C in 1 °C intervals over a 75 min period. Fluorescence values for the curve were normalized to the maximum and the minimum of the curve^33^.

### Protein crystallization

Crystals were grown by the sitting-drop vapour diffusion method at 20 °C from a protein concentration of 13 mg/mL^34^. The reservoir solution contained 2% (w/v) Tacsimate (pH 8.0), 0.1 M Tris (pH 8.5), and 16% (w/v) polyethylene glycol 3,350. Fully-grown crystals were obtained after 24 h of set-up. The crystals were cryo-protected in the reservoir solution supplemented with 20% (v/v) glycerol and flash-frozen in liquid nitrogen for data collection at 100 K.

### Data collection and processing

X-ray diffraction data were collected at the Advanced Photon Source beamline NE-CAT 24-ID-E using an ADSC Q315r detector and at the X25 beamline at the National Synchrotron Light Source at Brookhaven National Laboratory using a Pilatus 6 M detector. The diffraction images were processed and scaled with the HKL2000 package^35^.

### Structure determination and refinement

The structure of Tl-Cyn was solved by the molecular replacement method with the programme Phaser^36^, using the structure of *E. coli* cyanase (PDB code 1dw9) as the search model^20^. Manual model rebuilding was carried out with Coot^37^ and refinement with the programme Refmac^38^.

### Site directed mutagenesis

Site directed mutagenesis was carried out according to PCR-based methods. A set of overlapping oligonucleotide primer pairs with the desired mutations were designed to generate the mutant constructs and were synthesized from Integrated DNA Technologies (IDT), the sequences of which are shown in Supplementary Table S1. The recombinant plasmid (pET28a-*Tl-Cyn*) was used as a template for PCR amplification and the entire plasmid was amplified, using specific primers with the desired mutations (Supplementary Table S1). The PCR products were treated with *Dpn*I (methylation-dependent endonuclease) to remove the parent template and then transformed in *E. coli* DH5α. Further, plasmids were isolated from the transformed clones and verified by sequencing for successful mutagenesis. The mutants were expressed and purified following the same protocol as that for the wild-type protein.

### Cyanase assay

The cyanase assay was performed as previously described^25^. One unit (U) of cyanase is defined as the amount of enzyme that liberates 1 µmol of ammonium per minute under the defined assay conditions.

### Kinetic studies

The enzyme kinetics studies were performed by determining the velocities of the enzyme reactions at different concentrations of potassium cyanate (0.05 to 3.0 mM—Sigma). The apparent Michaelis constant (*K*_m_) and the maximal velocity (*V*_max_) of the enzyme activities were calculated by fitting the initial velocities and substrate concentrations into the Lineweaver–Burk plots. The catalytic efficiency of the enzyme was estimated as *k*_cat_/*K*_m_ ratio. Essentially the same values for *K*_m_ and *k*_cat_ were obtained from curve-fitting to the Michaelis–Menten equation (Supplementary Figs. S4, S5 and Supplementary Table S2).

### Inhibition kinetics by Formate and Malonate

To characterize the nature of the inhibition by formate and malonate, kinetic assays were carried out with varying concentrations of cyanate and bicarbonate along with different concentrations of inhibitors (one at a time). The inhibitory constants for uncompetitive and mixed inhibitions were analyzed by the Eqs. (1), (2) and (3), (4), respectively.

1 $$ {\text{Km}}_{{{\text{app}}}} = {{{\text{K}}_{{\text{m}}} } \mathord{\left/ {\vphantom {{{\text{K}}_{{\text{m}}} } {\left( {1 + {{[{\text{I}}]} \mathord{\left/ {\vphantom {{[{\text{I}}]} {{\text{K}}_{{{\text{ib}}}} }}} \right. \kern-\nulldelimiterspace} {{\text{K}}_{{{\text{ib}}}} }}} \right)}}} \right. \kern-\nulldelimiterspace} {\left( {1 + {{[{\text{I}}]} \mathord{\left/ {\vphantom {{[{\text{I}}]} {{\text{K}}_{{{\text{ib}}}} }}} \right. \kern-\nulldelimiterspace} {{\text{K}}_{{{\text{ib}}}} }}} \right)}} $$

2 $$ {\text{Vmax}}_{{{\text{app}}}} = {{{\text{Vmax}}} \mathord{\left/ {\vphantom {{{\text{Vmax}}} {\left( {1 + {{[{\text{I}}]} \mathord{\left/ {\vphantom {{[{\text{I}}]} {{\text{K}}_{{{\text{ib}}}} }}} \right. \kern-\nulldelimiterspace} {{\text{K}}_{{{\text{ib}}}} }}} \right)}}} \right. \kern-\nulldelimiterspace} {\left( {1 + {{[{\text{I}}]} \mathord{\left/ {\vphantom {{[{\text{I}}]} {{\text{K}}_{{{\text{ib}}}} }}} \right. \kern-\nulldelimiterspace} {{\text{K}}_{{{\text{ib}}}} }}} \right)}} $$

3 $$ {\text{Km}}_{{{\text{app}}}} = {{{\text{Km}}\left( {1 + {{[{\text{I}}]} \mathord{\left/ {\vphantom {{[{\text{I}}]} {{\text{K}}_{{{\text{ia}}}} }}} \right. \kern-\nulldelimiterspace} {{\text{K}}_{{{\text{ia}}}} }}} \right)} \mathord{\left/ {\vphantom {{{\text{Km}}\left( {1 + {{[{\text{I}}]} \mathord{\left/ {\vphantom {{[{\text{I}}]} {{\text{K}}_{{{\text{ia}}}} }}} \right. \kern-\nulldelimiterspace} {{\text{K}}_{{{\text{ia}}}} }}} \right)} {\left( {1 + {{[{\text{I}}]} \mathord{\left/ {\vphantom {{[{\text{I}}]} {{\text{K}}_{{{\text{ib}}}} }}} \right. \kern-\nulldelimiterspace} {{\text{K}}_{{{\text{ib}}}} }}} \right)}}} \right. \kern-\nulldelimiterspace} {\left( {1 + {{[{\text{I}}]} \mathord{\left/ {\vphantom {{[{\text{I}}]} {{\text{K}}_{{{\text{ib}}}} }}} \right. \kern-\nulldelimiterspace} {{\text{K}}_{{{\text{ib}}}} }}} \right)}} $$

4 $$ {\text{Vmax}}_{{{\text{app}}}} = {{{\text{Vmax}}} \mathord{\left/ {\vphantom {{{\text{Vmax}}} {\left( {1 + {{[{\text{I}}]} \mathord{\left/ {\vphantom {{[{\text{I}}]} {{\text{K}}_{{{\text{ib}}}} }}} \right. \kern-\nulldelimiterspace} {{\text{K}}_{{{\text{ib}}}} }}} \right)}}} \right. \kern-\nulldelimiterspace} {\left( {1 + {{[{\text{I}}]} \mathord{\left/ {\vphantom {{[{\text{I}}]} {{\text{K}}_{{{\text{ib}}}} }}} \right. \kern-\nulldelimiterspace} {{\text{K}}_{{{\text{ib}}}} }}} \right)}} $$

where Km_app_ is the apparent Michaelis constant (measured values of Km in the presence of the inhibitor), [I] the inhibitor concentration, K_ia_ and K_ib_ are the inhibitory constants for binding to the free enzyme and enzyme–substrate complex, respectively, and Vmax_app_ is the apparent maximal velocity (measured values of Vmax in the presence of inhibitor). SigmaPlot 10.0 was used to generate the plots.

### Sequence alignment

The alignment of selected sequences of cyanase was produced with Vector NTI Advance 11.5 and modified manually to include additional information.

## Supplementary Information

Supplementary Information.
