# Supplementary material for: Crystal structure of a thermophilic fungal cyanase and its implications on the catalytic mechanism for bioremediation
Source: Sci Rep. 2021 Jan 11;11:277. doi: 10.1038/s41598-020-79489-3 (PMC7801690; doi:10.1038/s41598-020-79489-3)
Supplement: Supplementary file 1 — Supplementary Information. [file 41598_2020_79489_MOESM1_ESM.pdf]

Supplementary Information

Crystal structure of a thermophilic fungal cyanase and its implications on the catalytic mechanism for bioremediation

Bibhuti Ranjan[1,2], Philip H. Choi[2], Santhosh Pillai[1], Kugenthiren Permaul[1], Liang Tong[1,2*] & Suren Singh[1*]

[1]Department of Biotechnology and Food Technology
Faculty of Applied Sciences
Durban University of Technology
Durban-4000, South Africa

[2]Department of Biological Sciences
Columbia University
New York, NY 10027, USA

Supplementary Figures

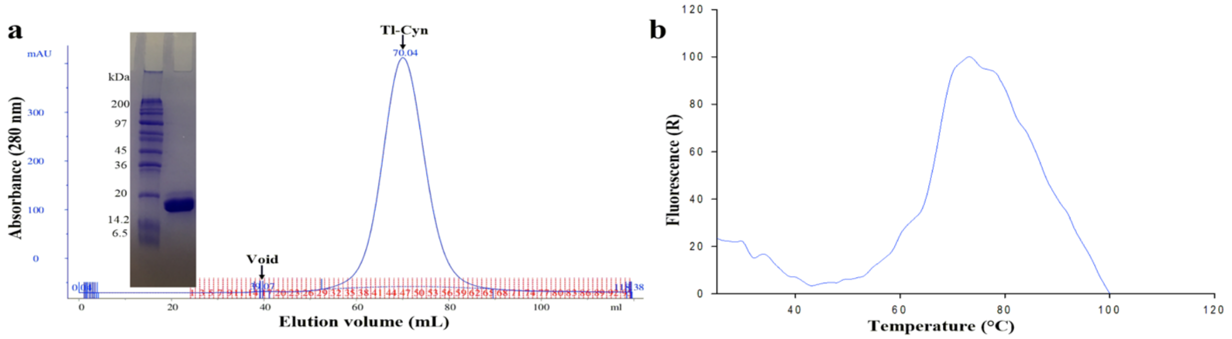

Supplementary Figure 1. Purification and thermal unfolding analysis of Tl-Cyn. a, Gel filtration chromatogram of the purified Tl-Cyn. Inset depicts SDS-PAGE image of a purified Tl-Cyn. **b,** Thermal shift assay to examine the thermostability of Tl-Cyn.

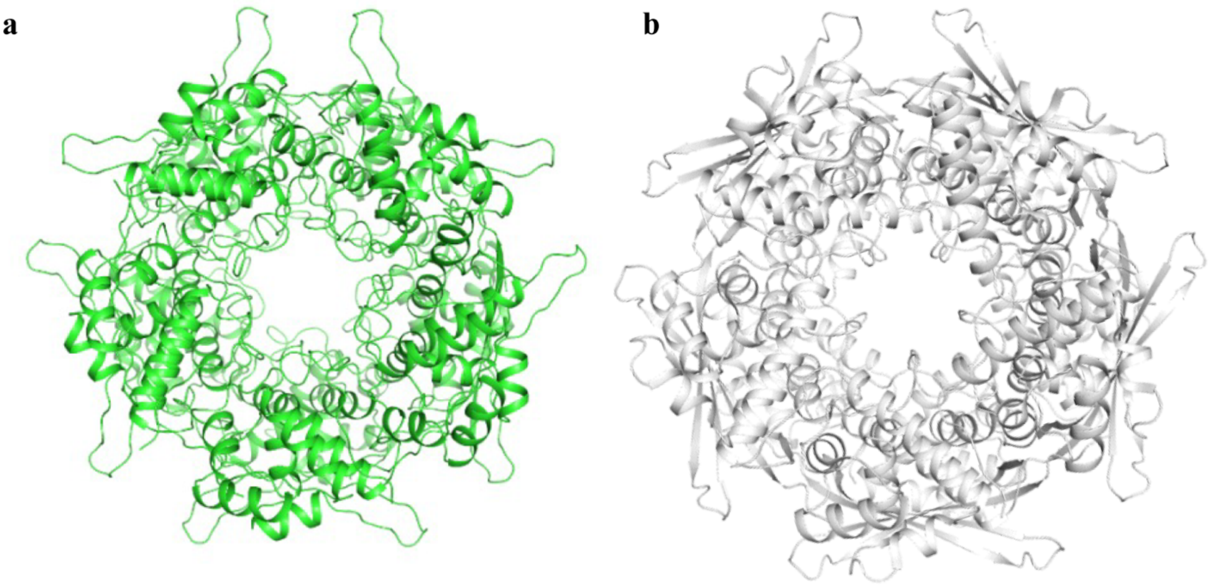

Supplementary Figure 2. Overall appearance of the two cyanase decamers. a Tl-Cyn. **b,** *E. coli*.

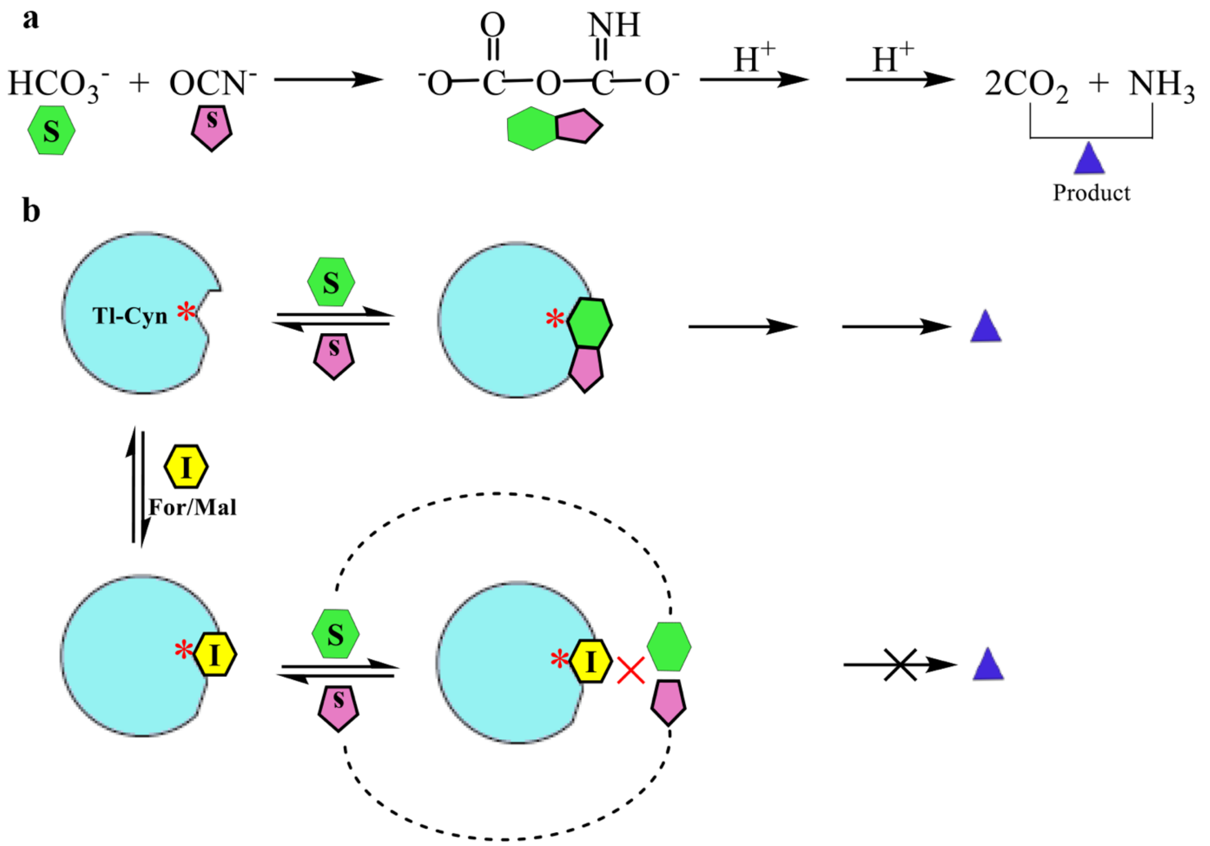

Supplementary Figure 3. Possible mechanism of the Tl-Cyn catalysis and inhibition. a, Schematic drawing for the mechanism of cyanate decomposition catalyzed by cyanase. The substrates are shown with different colors and shapes, bicarbonate green hexagon, and cyanate purple pentagon. **b,** A cartoon representation for the catalysis and kinetic inhibition of the Tl-Cyn. The active site is indicated with the red *asterisk*. The presence of the inhibitor at the active site of the Tl-Cyn has hindered substrate binding. The substrates are given separate colors as in **a**. The inhibitor molecule is shown in yellow hexagon.

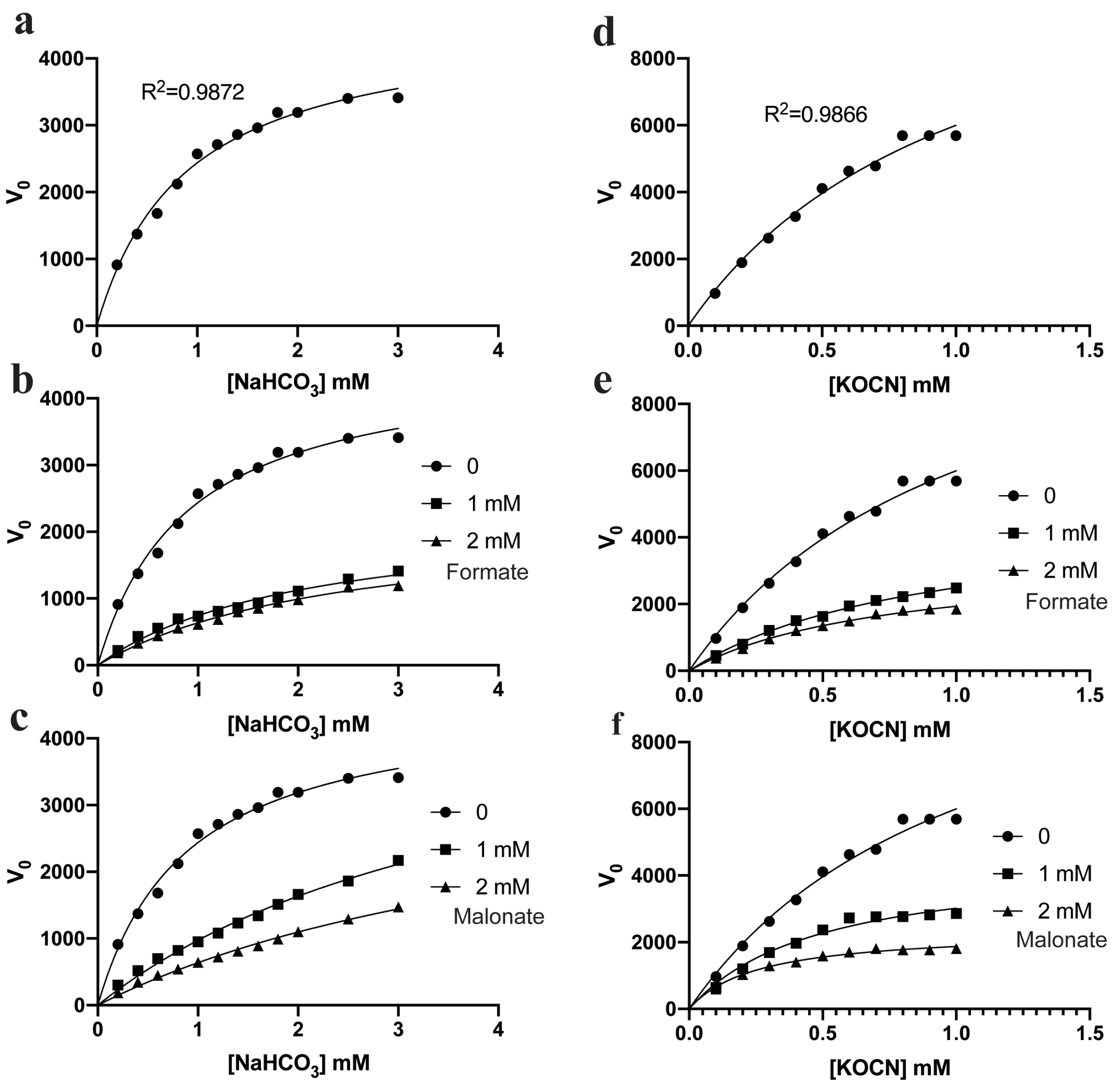

Supplementary Figure 4. Kinetic studies on the inhibition of Tl-Cyn by Non-linear curve-fitting using Michaelis-Menten plots. a, Titration of $NaHCO_3$, while keeping KOCN constant in the absence of inhibitors. **b,** Titration of $NaHCO_3$, while keeping KOCN constant in the presence of inhibitor (Formate). **c,** Titration of $NaHCO_3$, while keeping KOCN constant in the presence of inhibitor (Malonate). **d,** Titration of KOCN, while keeping $NaHCO_3$ constant in the absence of inhibitors. **e,** Titration of KOCN, while keeping $NaHCO_3$ constant in the presence of inhibitor (Formate). **f,** Titration of KOCN, while keeping $NaHCO_3$ constant in the presence of inhibitor (Malonate). The Non-linear curve-fittings and data prediction were performed using Prism 8 software.

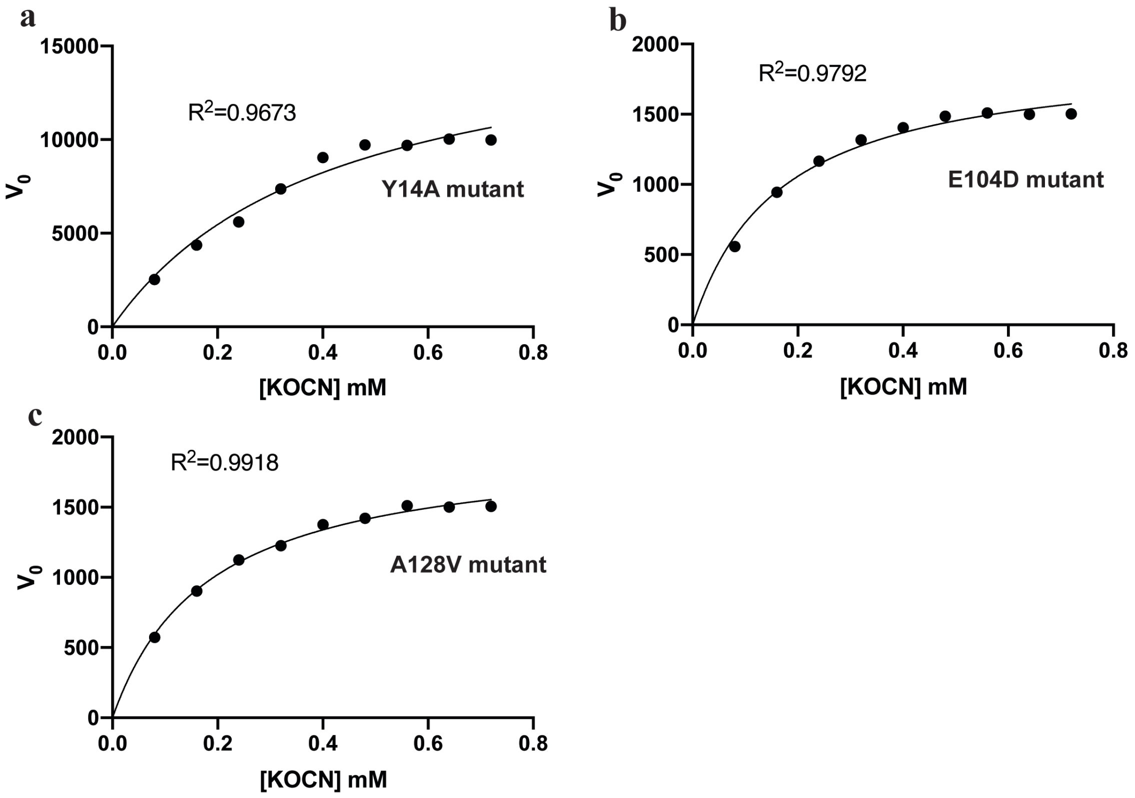

Supplementary Figure 5. Kinetic studies of Tl-Cyn mutants by Non-linear curve-fitting using Michaelis-Menten plots. a, Titration of KOCN, while keeping $NaHCO_3$ constant for Y14A mutant. **b,** Titration of KOCN, while keeping $NaHCO_3$ constant for E104D mutant. **c,** Titration of KOCN, while keeping $NaHCO_3$ constant for A128V mutant.

Supplementary Tables

Supplementary Table 1. Primer sequences for site directed mutagenesis

Primer	Sequence (5′→3′)*
BRF_Y14	GACGTCACTCAGCATCCA**GC**TCTACCCGCCTACTCCAA
BRR_Y14	TTGGAGTAGGCGGGTAGA**GC**TGGATGCTGAGTGACGTC
BRF_R101	AAGGAACCCTTGATTTAT**AA**ATTGTATGAGATTGTGCA
BRR_R101	TGCACAATCTCATACAAT**TT**ATAAATCAAGGGTTCCTT
BRF_E104	GATTTATCGATTGTATGA**C**ATTGTGCAGAATTATGGA
BRR_E104	TCCATAATTCTGCACAAT**G**TCATACAATCGATAAATC
BRF_S127	TCGGGGACGGTATCATGA**C**TGCGATCAGCTTTTCAAC
BRR_S127	GTTGAAAAGCTGATCGCA**G**TCATGATACCGTCCCCGA
BRF_A128	GGGACGGTATCATGAGTG**T**GATCAGCTTTTCAACGTC
BRR_A128	GACGTTGAAAAGCTGATC**A**CACTCATGATACCGTCCC

*Substituted nucleotides are indicated in bold.

Supplementary Table 2. Summary of kinetic data for wild-type and mutant cyanases from Non-linear curve-fitting using Michaelis-Menten plots

Protein	k_{cat} (s^{-1})	K_m (mM) (for cyanate)	k_{cat}/K_m (s^{-1} M^{-1})
Wild-type	3.52×10^4	0.72 (from 95% CI)	4.88×10^7
Y14A	4.78×10^4	0.41 (from best-fit)	11.66×10^7
R101K	NA[α]	NA[α]	NA[α]
E104D	5.51×10^3	0.17 (from best-fit)	3.24×10^7
S127T	NA[α]	NA[α]	NA[α]
A128V	5.54×10^3	0.18 (from best-fit)	3.08×10^7
A128V/ R101K	NA[α]	NA[α]	NA[α]

[α]No activity was observed under the same conditions.
